# Transperineal versus Transrectal MRI/TRUS fusion-guided prostate biopsy in a large, ethnically diverse, and multiracial cohort

**DOI:** 10.1590/S1677-5538.IBJU.2024.0354

**Published:** 2024-07-25

**Authors:** Lorenzo Storino Ramacciotti, David Strauss, Francesco Cei, Masatomo Kaneko, Daniel Mokhtar, Jie Cai, Delara Jadvar, Giovanni E. Cacciamani, Manju Aron, Pierre B. Halteh, Vinay Duddalwar, Inderbir Gill, Andre Luis Abreu

**Affiliations:** 1 University of Southern California Keck School of Medicine USC Institute of Urology Los Angeles California USA USC Institute of Urology, Keck School of Medicine, University of Southern California, Los Angeles, California, USA; 2 University of Southern California Keck School of Medicine Center for Image-Guided Surgery, Focal Therapy and Artificial Intelligence for Prostate Cancer Los Angeles California USA Center for Image-Guided Surgery, Focal Therapy and Artificial Intelligence for Prostate Cancer, Keck School of Medicine, University of Southern California, Los Angeles, California, USA; 3 University of Southern California Department of Radiology Keck School of Medicine Los Angeles California USA Department of Radiology Keck School of Medicine, University of Southern California, Los Angeles, California, USA; 4 University of Southern California Department of Pathology Keck School of Medicine Los Angeles California USA Department of Pathology Keck School of Medicine, University of Southern California, Los Angeles, California, USA

**Keywords:** Prostatic Neoplasms, Ultrasound, High-Intensity Focused, Transrectal, Ethnicity, Magnetic Resonance Imaging

## Abstract

**Purpose:**

To compare transperineal (TP) vs transrectal (TR) magnetic resonance imaging (MRI) and transrectal ultrasound (TRUS) fusion-guided prostate biopsy (PBx) in a large, ethnically diverse and multiracial cohort.

**Materials and Methods:**

Consecutive patients who underwent multiparametric (mp) MRI followed by TP or TR TRUS-fusion guided PBx, were identified from a prospective database (IRB #HS-13-00663). All patients underwent mpMRI followed by 12-14 core systematic PBx. A minimum of two additional target-biopsy cores were taken per PIRADS≥3 lesion. The endpoint was the detection of clinically significant prostate cancer (CSPCa; Grade Group, GG≥2). Statistical significance was defined as p<0.05.

**Results:**

A total of 1491 patients met inclusion criteria, with 480 undergoing TP and 1011 TR PBx. Overall, 11% of patients were Asians, 5% African Americans, 14% Hispanic, 14% Others, and 56% White, similar between TP and TR (p=0.4). For PIRADS 3-5, the TP PBx CSPCa detection was significantly higher (61% vs 54%, p=0.03) than TR PBx, but not for PIRADS 1-2 (13% vs 13%, p=1.0). After adjusting for confounders on multivariable analysis, Black race, but not the PBx approach (TP vs TR), was an independent predictor of CSPCa detection. The median maximum cancer core length (11 vs 8mm; p<0.001) and percent (80% vs 60%; p<0.001) were greater for TP PBx even after adjusting for confounders.

**Conclusions:**

In a large and diverse cohort, Black race, but not the biopsy approach, was an independent predictor for CSPCa detection. TP and TR PBx yielded similar CSPCa detection rates; however the TP PBx was histologically more informative.

## INTRODUCTION

An accurate prostate cancer (PCa) diagnosis relies on a quality prostate biopsy (PBx) followed by histological evaluation ( 1 ). The European Association of Urology guidelines strongly recommend the transperineal (TP) approach over the transrectal (TR) approach as the gold standard for prostate biopsy. This recommendation is based on observational studies and meta-analyses showing lower rates of infectious complications and hospital re-admissions for sepsis ( 2 , 3 ). In contrast, the American Urological Association guidelines state that clinicians may choose either a TR or TP biopsy approach ( 4 ). One meta-analysis suggested higher PCa and clinically significant PCa (CSPCa) detection rates using the TP approach, especially for anterior lesions ( 5 ), while another stated no differences between approaches ( 6 ).

Randomized clinical trials comparing both approaches have recently been published, evidencing similar cancer detection and complication rates ( 7 – 10 ). However, these trials’ cohorts consisted mostly of white patients, limiting the findings’ applicability to other racial and ethnic groups. We hypothesized that PCa detection rates on different PBx approaches might be impacted by the patient's race and ethnicity.

Hence, this study aimed to compare magnetic resonance imaging (MRI) transrectal ultrasound (TRUS) fusion-guided TP and TR PBx cancer detection rates, histologic findings, and periprocedural outcomes in a multiracial and ethnically diverse cohort.

## MATERIAL AND METHODS

### Study design and population

Consecutive patients who underwent multiparametric (mp) MRI followed by TP or TR TRUS-fusion guided PBx, between January 2016 and May 2023, were identified from a prospective institutional database (IRB #HS-13-00663). Inclusion criteria were: I) patients who underwent mpMRI within 6 months of biopsy: II) patients who underwent MRI/TRUS fusion TP or TR PBx. Exclusion criteria were: I) any prior treatment for PCa; II) any prior prostate surgery; III) saturation biopsies; IV) patients with mpMRI that didn't meet Prostate Imaging Reporting & Data System (PIRADS) standards ( 11 , 12 ).

### MRI acquisition and interpretation

The mpMRIs (T2W, DWI, ADC and DCE) were acquired and interpreted in accordance with the relevant PIRADS version (2.0 or 2.1) ( 11 , 12 ) prevalent during the biopsy timeframe, as previously described ( 13 – 17 ). Images were interpreted by radiologists with over 5 years of expertise in prostate mpMRI reading. The lesion with the highest PIRADS score, followed by the largest dimension, was defined as the index lesion.

### Prostate biopsy protocol

**Figure 1:**
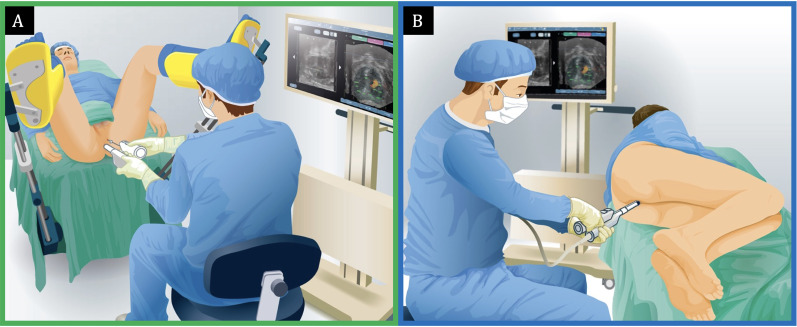
Prostate biopsy setup and templates. A) MRI/TRUS fusion-guided free-hand transperineal prostate biopsy. In this procedure, the biopsy gun is inserted through a coaxial needle, minimizing multiple punctures through the perineal skin. The patient remains in the supine position during the entire procedure. B) MRI/TRUS fusion-guided transrectal prostate biopsy. The prostate is sampled by inserting the needle through the rectum, with the patient placed in the left lateral decubitus position.

**Figure 2:**
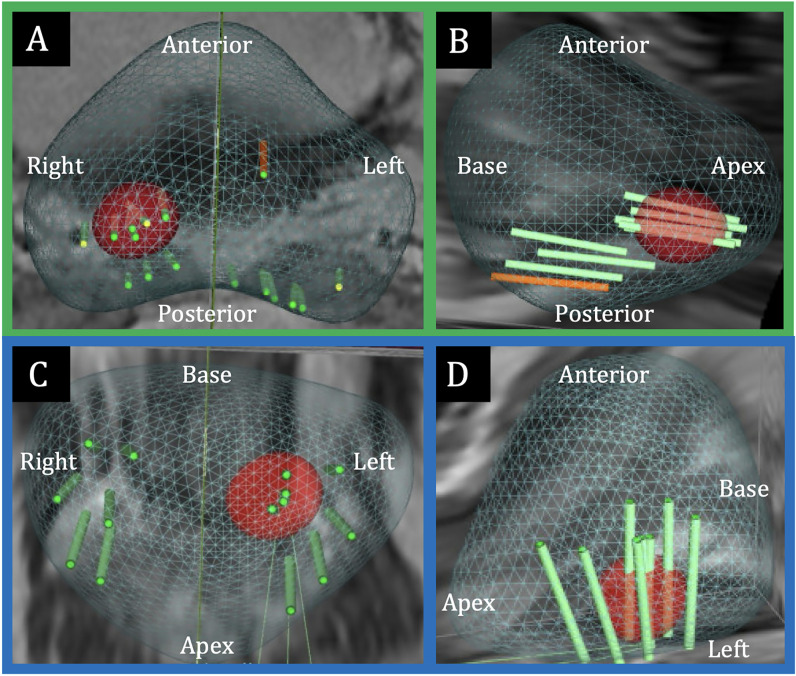
Prostate biopsy templates. A) Axial and B) right sagittal view of the transperineal MRI/TRUS fusion-guided prostate biopsy template. C) Coronal and D) left sagittal view of the transrectal MRI/TRUS fusion-guided prostate biopsy template.

Prostate biopsies were performed transperineally or transrectally by a single urologist (ALA) ( Figure-1 ). All procedures were performed using a three-dimensional organ-tracking elastic image fusion system (Trinity, Koelis®, France) and an 18G needle-biopsy, as previously described ( 13 – 19 ). All patients underwent mpMRI followed by a 12–14 core systematic biopsy (SB), with at least two additional target biopsy (TB) core samples per suspicious lesion for patients with PIRADS 3-5 lesions ( Figure-2 ). TP and TR PBx were routinely offered and performed under local anesthesia in the outpatient clinic. However, PBx were eventually performed under sedation in the operating room based on patient preference or for the initial TP PBx cases. The PBx specimens were individually labeled and submitted in separate containers for uropathologist evaluation according to the International Society of Urological Pathology (ISUP) guidelines ( 20 ).

Empiric antibiotic prophylaxis was prescribed according to American Urological Association recommendations ( 21 ). Patients undergoing TR PBx received 3 days of Ciprofloxacin, Bactrim, or Cefuroxime with augmentation of Gentamicin IM at the time of biopsy. Povidone-iodine preparation was not performed before TR or TP PBx. Not all patients undergoing TP PBx received antibiotic prophylaxis ( 22 ). When the TP PBx was integrated into our institution's clinical practice, between 2016 and 2017, there was insufficient evidence supporting an antibiotic-free procedure at that time. The initial patients received a single dose of Cefuroxime 500mg ( 21 , 23 ). However, with the emergence of new level 1 evidence supporting the safety of performing TP PBx without antibiotic prophylaxis, only specific subsets of patients continued to receive antibiotic prophylaxis thereafter. These included patients with cardiac valve disease or replacement, those with a history of acute prostatitis, or those who were immunosuppressed.

### Transperineal and transrectal approach

TR approach was offered to all initial patients as the operator did not perform TP PBx in 2016. With the adoption of TP PBx in 2017, patients were offered either the TP or TR approach based on their risk of complications. Specifically, patients with an increased risk of rectal bleeding or urinary tract infections were recommended the TP PBx. Patients with an increased risk of rectal bleeding included those on antiplatelet or anticoagulant therapy or those with a previous history of rectal bleeding on prior PBx. MRI interpretation and lesion location did not define the approach to be used. As of 2023, the operator had transitioned to performing TP PBx, with the TR approach being reserved for cases based on patient's request.

### Endpoints and definitions

The primary endpoint was the detection of clinically significant PCa (CSPCa), defined as grade group (GG) ≥ 2 on SB, TB, and SB plus TB. Secondary outcomes included: I) detection of high-grade PCa, defined as GG ≥ 3; II) amount of PCa core length and percent of core involvement by PCa on TB; III) periprocedural outcomes.

The index lesion was defined as the highest PIRADS score, followed by the largest dimension. The location of the index lesion was categorized based on its position in the prostate on MRI: anterior index lesions were defined as those located from the 9:00 to 3:00 o'clock positions, while posterior lesions were located from 3:00 to 9:00 o'clock positions. If the largest lesion spanned both anterior and posterior locations, it was assigned to both locations. This classification was also applied to lesions located at the mid, base, and apex of the prostate. Prostate volume (PV) was estimated based on MRI measurements using the ellipsoid formula ( PV=height×width×length×0.52 ).

Complications were recorded up to 30 days post-biopsy using the Clavien-Dindo grading system ( 24 , 25 ). Procedure time was recorded from the instant the ultrasound probe was introduced into the patient's rectum to the moment it was removed. Recorded pain levels were all self-reported immediately after the procedure and exclusive to cases done under local anesthesia ( 18 ). Patients were asked to rate on a visual analog scale (0–10) the overall pain experienced throughout the procedure. Baseline demographics and MRI details were also analyzed.

Race and ethnicity were self-reported by patients and defined according to the NIH reporting standards as follows: Hispanic or Latino (Latino), non-Hispanic Asian (Asian), non-Hispanic Black or African American (Black), non-Hispanic White (White), and Other. The Other category includes patients who didn't report or identify as any race or ethnicity, American Indian or Alaska Native, and Native Hawaiian or Other Pacific Islander due to small sample sizes ( 26 ).

### Statistical Analysis

The statistical software package SAS version 9.4 (SAS Institute Inc., Cary, NC, USA) was used for all analyses in this study. Patients were divided into two separate cohorts of PIRADS 1-2 (negative MRI) and PIRADS 3-5 for sub-group analysis. The Wilcoxon rank sum test was used for continuous variables, and Pearson's chi-square was used for categorical variables. Univariable and multivariable logistic and linear regressions were performed to model the dichotomous and continuous outcomes, respectively, to identify clinical parameters related to CSPCa, high-grade PCa detection, and PBx histolologic findings. MRI lesion location was divided into "anterior versus non-anterior lesions" and "apical versus non-apical lesions" for univariable and multivariable analyses. A two-sided p-value of < 0.05 was considered statistically significant.

## RESULTS

A total of 1491 patients met the inclusion criteria, with 480 undergoing TP and 1011 TR PBx. Overall, 11% of patients were Asian, 5% Black or African American, 14% Latino, 14% Others, and 56% White, similar between TP and TR (p=0.41). The median age (67 vs 66Y, p=0.048), PSA (6.8 vs 6.5ng/mL, p=0.09), PSA density (0.13 vs 0.12ng/mL2, p=0.27), prostate volume (55 vs 52cc, p=0.53), and PIRADS distribution (PIRADS 3-5, 75% vs 74%, p=0.75) were similar between the TP and TR groups, respectively ( Table-1 ). The median MRI index lesion size (14 vs 12mm, p=0.001) and number of MRI lesions (1 vs 1; p=0.009) were higher for the TP group.

**Table 1:** Baseline characteristics of the transperineal and transrectal cohorts.

MRI									
	All patients			PIRADS 1-2			PIRADS 3-5		
	Perineal	Rectal	p	Perineal	Rectal	p	Perineal	Rectal	p
**No. of Patients, n (%)**	480	1011		122	265		358	746	
**Age, year, median (IQR)**	67 (62-72)	66 (61-70)	0.048	65 (60-69)	64 (59-69)	0.3	67 (62-72)	66 (61-71)	0.09
**Carlson comorbidity index, median (IQR)**	1 (0-2)	1 (0-2)	0.9	0 (0-2)	1 (0-2)	0.5	1 (0-2)	1 (0-2)	0.8
**Family History PCa, n (%)**	122 (27.5)	261 (27.8)	0.9	37 (33.0)	64 (25.7)	0.16	85 (25.7)	197 (28.5)	0.4
**Race, n (%)**			0.4			0.07			0.8
**White**	265 (55.2)	571 (56.5)		62 (50.8)	157 (59.3)		203 (56.7)	414 (55.5)	
**Black**	25 (5.2)	48 (4.8)		8 (6.6)	18 (6.8)		17 (4.8)	30 (4.0)	
**Latino**	67 (14.0)	147 (14.5)		19 (15.6)	34 (12.8)		48 (13.4)	113 (15.2)	
**Asian**	63 (13.1)	101 (10.0)		22 (18.0)	23 (8.7)		41 (11.5)	78 (10.5)	
**Other or not reported**	60 (12.5)	144 (14.2)		11 (9.0)	33 (12.5)		49 (13.7)	111 (14.9)	
**Biopsy History, n (%)**			<0.001			0.002			0.001
**Naïve**	311 (65.6)	524 (51.9)		78 (65.6)	124 (46.8)		233 (65.6)	400 (53.8)	
**Negative**	91 (19.2)	254 (25.2)		26 (21.9)	75 (28.3)		65 (18.3)	179 (24.1)	
**In active surveillance**	72 (15.2)	231 (22.9)		15 (12.6)	66 (24.9)		57 (16.1)	165 (22.2)	
**PSA, ng/mL, median (IQR)**	6.8 (4.9-10.3)	6.5 (4.8-9.7)	0.09	6.9 (5.2-9.9)	6.2 (4.5-8.5)	0.02	6.7 (4.9-10.5)	6.6 (4.9-10)	0.5
**PSA density, ng/mL^2^, median (IQR)**	0.13 (0.08-0.20)	0.12 (0.08-0.20)	0.3	0.11 (0.08-0.17)	0.10 (0.07-0.14)	0.048	0.13 (0.09-0.22)	0.13 (0.08-0.22)	0.9
**Suspicion for PCa on DRE, n (%)**	113 (23.5)	265 (26.2)	0.3	17 (13.9)	44 (16.6)	0.5	96 (26.8)	221 (29.6)	0.3
**Clinical T stage, n (%)** *			0.19			0.8			0.2
**T1**	375 (80.5)	784 (78.6)		111 (94.1)	244 (92.4)		264 (75.9)	540 (73.6)	
**T2**	62 (27.3)	165 (16.5)		6 (5.1)	18 (6.8)		56 (16.1)	147 (20.0)	
**T3/T4**	29 (6.2)	49 (4.9)		1 (0.8)	2 (0.8)		28 (8.0)	47 (6.4)	
**Prostate Volume, cc, median (IQR)**	55 (38-77)	52 (37-75)	0.53	62 (42-86)	59 (43-86)	0.91	52 (37-72)	49 (36-72)	0.37
**No. MRI lesions, median (IQR)**	1 (1-1)	1 (1-2)	0.23	0 (0)	0 (0)	-	1 (1-2)	1 (1-2)	0.009
**MRI index lesion location, n (%)**				-	-	-			
**Anterior**	148 (30.8)	211 (20.9)	<0.001				146 (40.8)	206 (27.6)	<0.001
**Posterior**	264 (55)	572 (56.6)	0.58	-	-	-	256 (71.5)	561 (75.2)	0.21
**MRI index lesion location** ** **, n (%)**									
**Base**	69 (14.4)	159 (15.7)	0.54	-	-	-	65 (18.2)	152 (20.4)	0.42
**Mid**	190 (39.6)	318 (31.5)	0.002	-	-	-	184 (51.4)	304 (40.8)	0.001
**Apex**	87 (18.1)	186 (18.4)	0.94	-	-	-	87 (24.3)	179 (24)	0.94
**MRI index lesion size** ** **, mm, median (IQR)**	14 (10-19)	12 (9-17)	0.001	-	-	-	14 (10-19)	12 (9-17)	0.001
**PIRADS score, n (%)**			0.75			-			-
**PIRADS 1-2**	122 (25.4)	265 (26.2)		122 (100)	265 (100)		-	-	
PIRADS 3-5*	358 (74.6)	746 (73.8)		-	-		358 (100)	746 (100)	
**PIRADS 3**	93 (19.4)	330 (32.6)		-	-		93 (26)	330 (44.2)	
**PIRADS 4**	139 (29)	271 (26.8)		-	-		139 (38.8)	271 (36.3)	
**PIRADS 5**	126 (26.3)	145 (14.3)		-	-		126 (35.2)	145 (19.4)	

PIRADS = Prostate Imaging Reporting and Data System; MRI = magnetic resonance imaging; No., number; IQR = Interquartile Range; PCa = prostate cancer; CSPCa = Clinically significant PCa (Grade Group > 1); DRE = digital rectal examination; Anterior lesion on MRI 9-3:00 position; otherwise it's posterior.

* DRE findings of a possible clinical stage in case prostate biopsy confirms cancer.

** Index lesion (highest PIRADS, then the largest lesion)

### Primary Endpoint

Overall, for all patients, TP detected more CSPCa (48.8% vs 43.1%, p=0.04) compared to the TR PBx on SB plus TB. For PIRADS 3-5, TP detected more CSPCa (60.9% vs 53.9%, p=0.03) on SB plus TB. For PIRADS 1-2, CSPCa detection was not different between biopsy approaches (13.1% vs 12.8%, p=1.0). CSPCa detection rates for anterior (64.2% vs 57.8%, p=0.2) and non-anterior (41.9% vs 39.2%, p=0.4) lesions on MRI were similar between TP and TR PBx, respectively. Detailed biopsy outcomes are reported in Table-2 . On a multivariable logistic regression model, age, negative biopsy history, PSA, Black race, suspicious digital rectal examination, PV, PIRADS 3-5, and the number of TB cores taken were independent predictors for CSPCa detection ( Table-3 ). Although the biopsy approach, anterior and non-apical lesions on MRI were independent predictors for CSPCa detection in the univariable analysis, they were not independent predictors in the multivariable analysis.

**Table 2:** Outcomes of transperineal vs transrectal MRI/TRUS fusion prostate biopsy.

		MRI					
		PIRADS 1-2			PIRADS 3-5		
		Perineal	Rectal	p	Perineal	Rectal	p
**No. of Patients, n (%)**		122	265		358	746	
**Prostate Biopsy Pathology**							
**Grade Group**				0.4			0.09
	Benign	80 (65.6)	176 (66.4)		101 (28.2)	241 (32.3)	
	1	26 (21.3)	55 (20.8)		39 (10.9)	103 (13.8)	
	2	11 (9.0)	24 (9.0)		89 (24.9)	193 (25.9)	
	3	1 (0.8)	7 (2.6)		52 (14.5)	100 (13.4)	
	4	2 (1.6)	2 (0.8)		42 (11.7)	59 (7.9)	
	5	2 (1.6)	1 (0.4)		35 (9.8)	50 (6.7)	
**No. of TB cores taken, median (IQR)**		-	-	-	4 (4-6)	4 (2-5)	<0.001
**No. of positive TB cores, median (IQR)**		-	-	-	3 (0-5)	1 (0-3)	<0.001
**PCa detection SB + TB, N (%)**		42 (34.4)	89 (33.6)	0.9	257 (71.8)	505 (67.7)	0.19
**PCa detection SB, N (%)**		42 (34.4)	89 (33.6)	0.9	208 (58.1)	471 (63.1)	0.11
**PCa detection TB, N (%)**		-	-	-	233 (65.1)	406 (54.4)	<0.001
**CSPCa SB + TB, N (%)**		16 (13.1)	34 (12.8)	1.0	218 (60.9)	402 (53.9)	0.03
**CSPCa SB, N (%)**		16 (13.1)	34 (12.8)	1.0	162 (45.3)	361 (48.4)	0.3
**CSPCa TB, N (%)**		-	-	-	199 (55.6)	301 (40.4)	<0.001
**GG≥3 detection SB + TB, N (%)**		5 (4.1)	10 (3.8)	0.9	129 (36)	209 (28)	0.008
**GG≥3 detection SB, N (%)**		5 (4.1)	10 (3.8)	0.9	89 (24.9)	176 (23.6)	0.6
**GG≥3 detection TB, N (%)**		-	-	-	116 (32.4)	145 (19.4)	<0.001
**CSPCa SB + TB, N (%)**							
	PIRADS 3	-	-	-	28 (30.1)	104 (31.5)	0.9
	PIRADS 4	-	-	-	85 (61.2)	174 (64.2)	0.6
	PIRADS 5	-	-	-	105 (83.3)	124 (85.5)	0.7
**CSPCa TB per lesion, N (%)**							
	PIRADS 3	-	-	-	20 (21.5)	60 (18.2)	0.4
	PIRADS 4	-	-	-	80 (57.6)	136 (50.2)	0.17
	PIRADS 5	-	-	-	99 (78.6)	105 (72.4)	0.3
**GG≥3 SB + TB, N (%)**							
	PIRADS 3	-	-	-	10 (10.8)	44 (13.3)	0.6
	PIRADS 4	-	-	-	46 (33.1)	77 (28.4)	0.4
	PIRADS 5	-	-	-	73 (57.9)	88 (60.7)	0.7
**GG≥3 TB per lesion, N (%)**							
	PIRADS 3	-	-	-	8 (8.6)	19 (5.8)	0.3
	PIRADS 4	-	-	-	41 (29.5)	56 (20.7)	0.0502
	PIRADS 5	-	-	-	67 (53.2)	70 (48.3)	0.5
**Maximum cancer core length SB + TB (mm), median (IQR)**		2 (1-6)	3 (1-6)	0.5	11 (7-13)	8 (5-12)	<0.001
**Maximum cancer core length SB (mm), median (IQR)**		2 (1-6)	3 (1-6)	0.5	7 (4-11)	6 (3-9)	0.008
**Maximum cancer core length TB (mm), median (IQR)**		-	-	-	11 (7-13)	8 (5-11)	<0.001
**Maximum cancer core percent SB + TB (%), median (IQR)**		10 (7-43)	20 (5-40)	0.9	80 (60-95)	60 (30-80)	<0.001
**Maximum cancer core percent SB (%), median (IQR)**		10 (7-43)	20 (5-40)	0.9	60 (30-80)	40 (20-70)	<0.001
**Maximum cancer core percent TB (%), median (IQR)**		-	-	-	80 (60-91)	60 (30-80)	<0.001

TRUS = transrectal ultrasound; PIRADS = Prostate Imaging Reporting and Data System; MRI = magnetic resonance imaging; No. = number; IQR, Interquartile Range; PCa = prostate cancer; CSPCa = Clinically significant PCa (Grade Group > 1); SB = systematic biopsy; TB = target biopsy.

**Table 3:** Univariable and Multivariable logistic regression analyses for clinically significant cancer detection on transperineal versus transrectal MRI/TRUS fusion prostate biopsy.

Variables		Univariate			Multivariate		
		OR	CI (95%)	p	OR	CI (95%)	p
**Age, year**		1.06	1.04-1.07	<0.001	1.06	1.04-1.08	<0.001
**Family History PCa**		1.02	0.80-1.29	0.87			
**Biopsy history**				<0.001			<0.001
	Previous Negative biopsy vs Naïve	0.48	0.37-0.62		0.58	0.41-0.80	
	Previous Positive biopsy vs Naïve	1.03	0.79-1.34		1.17	0.84-1.63	
**PSA, ng/mL**		1.05	1.03-1.07	<0.001	1.08	1.05-1.11	<0.001
**PSA density** * , **ng/mL** ^2^		1.07	1.06-1.08	<0.001			
**Race**				0.16			0.01
	Asian vs NH-White	0.84	0.60-1.18		0.68	0.44-1.05	
	Hispanic vs NH-White	0.86	0.64-1.17		0.91	0.63-1.35	
	Black vs NH-White	1.60	0.99-2.63		2.49	1.31-4.74	
	Others vs NH-White	0.90	0.66-1.23		0.81	0.55-1.19	
**DRE, suspicious vs non-suspicious**		4.1	3.20-5.29	<0.001	3.18	2.3-4.4	<0.001
**Prostate Volume, cc**		0.983	0.980-0.987	<0.001	0.979	0.974-0.983	<0.001
**No. MRI lesions**		1.92	1.69-2.19	<0.001			
**MRI lesion size, mm**		1.06	1.04-1.08	<0.001			
**PIRADS 3-5 vs PIRADS 1-2**		8.63	6.33-12.01	<0.001	4.74	2.99-7.60	<0.001
**MRI lesion location, n (%)**							
	Non-anterior vs anterior	0.43	0.34-0.55	<0.001	0.90	0.66-1.23	0.5
**MRI lesion location, n (%)**							
	Non-apical vs apex	0.72	0.55-0.93	0.01	1.29	0.92-1.80	0.13
**No. TB cores taken**		1.35	1.29-1.41	<0.001	1.15	1.08-1.24	<0.001
**Prostate biopsy approach TP vs TR**		1.25	1.01-1.56	0.04	1.04	0.78-1.39	0.78

PIRADS = Prostate Imaging Reporting and Data System; MRI = magnetic resonance imaging; OR = odds ratio; CI, confidence interval; PCa = prostate cancer; CSPCa = Clinically significant PCa (Grade Group ≥ 2); DRE = digital rectal examination; DRE = digital rectal examination; NH = non-Hispanic.

* PSA density was calculated per 0.01 unit.

### Secondary outcomes

TP detected more high-grade PCa (27.9% vs 21.7%, p=0.009) in the overall cohort and in the PIRADS 3-5 subgroup (36% vs 28%, p=0.008) on SB plus TB. For PIRADS 1-2, high-grade PCa detection rates (4.1% vs 3.8%, p=0.9) were not different between TP and TR, respectively. On a multivariable logistic regression model for GG≥3 PCa detection, age, PSA, suspicious digital rectal examination, PV, PIRADS 3-5, and non-apical lesions were independent predictors ( **Supplementary Table-1, see more** ). The biopsy approach and anterior lesion on MRI were not independent predictors for high-grade PCa detection.

TP PBx had a higher median maximum TB cancer core length (11 vs 8 mm, p<0.001) and percent involvement by cancer (80% vs 60%, p<0.001) than the TR PBx. On multivariable linear regressions, TP PBx was still an independent predictor for higher cancer core length and percent involvement on TB ( **Supplementary Tables 2 and 3, see more** ).

Median procedure time was longer for TP PBx (20 vs 19 min, p<0.001) ( **Supplementary Table-4, see more** ). The median patient self-reported pain levels were similar between the biopsy approaches (TP 3 vs TR 4, p=0.6). The 30-day complications were low and similar (1.9% vs 1.7%, p=0.8) between the TP and TR groups, respectively ( **Supplementary Table-4, see more** ). Four patients in the TR group and one patient in the TP group developed sepsis. Clavien-Dindo Grade ≥III complications occurred in 2 (0.4%) and 3 (0.3%) of patients in the TP and TR groups (p=0.9), respectively.

## DISCUSSION

In this single-center prospective database study, TP PBx detected more CSPCa and high-grade PCa than TR PBx in a large ethnically diverse and multiracial cohort in univariable analysis; however, the biopsy approach was not an independent predictor on multivariable analyses. The TP approach yielded higher cancer core length and percent involvement in TB and was an independent predictor on multivariable analyses. The median procedure time was lower for the TR group, while patients’ self-reported pain levels and complications were similar between both approaches. Hence, while being similar in safety and tolerance, TP PBx is potentially histologically more informative than TR PBx.

Recent randomized trials have compared the TP and TR approaches. Mian et al. assessed the efficacy and complications between TP and TR in a single center setting in the US ( 8 , 9 ). CSPCa detection rates (43.2% vs 47.1; odds ratio [OR], 1.17; 95% Confidence Interval [CI], 0.88-1.55) and complications (2.7% vs 2.6%, OR, 1.06; 95% CI, 0.43 to 2.65; p=.99) were similar for TP and TR, respectively. No patients in either group developed sepsis. Similarly, in Hu et al. PREVENT trial ( 7 ), CSCPa was detected in 53% and 50% of patients for TP and TR PBx, respectively (adjusted difference 2.0%; 95% CI −6.0%, 10%). No significant difference in infectious complications was noted between approaches (difference, −1.4%; Newcombe hybrid score 95% CI −0.3, 3.2; p = 0.059), although no cases of infection occurred in the TP arm. Ploussard et al. trial ( 10 ) reported no statistically significant difference in CSPCa detection rates for MRI-targeted TP or TR PBx (47.2% vs 54.2%, p=0.62).

However, a major limitation is the patient selection in these trials, which consists overwhelmingly of white males. Homogeneous patient populations limit the external validity of the results, thereby potentially leading to inadequate management of PCa ( 27 , 28 ). For instance, Black men tend to present with more aggressive prostate cancer at initial diagnosis, are less likely to receive definitive treatment, and have a higher incidence and mortality rate from prostate cancer compared to other races ( 29 ). Hence the importance of accurate early detection in this population. In contrast, it has been suggested that Asian men are less likely to be diagnosed with prostate cancer compared to white men, independent of PSA levels, suggesting that biological, genetic, or environmental factors may influence the disease's development ( 30 ). Therefore, a strength of this study is having assessed the PBx approach comparison in a diverse cohort including Asians, Black, Latino, White and Other patients.

The higher detection rates of CSPCa and high-grade PCa in the univariable analysis for the TP group can be attributed to a higher number of lesions on MRI and greater lesion sizes among these patients. To address this imbalance, multivariable logistic regression analyses were conducted. Interestingly, TP patients had more anterior lesions compared to TR patients, despite biopsy approach selection not being based on lesion location. In the trial by Ploussard et al. ( 10 ), MRI-targeted TR significantly detected more posterior CSPCa than the TP approach (59.0% vs. 44.3%, p=0.04), while TP detected more CSPCa in anterior lesions, although this was not statistically significant (40.6% vs. 26.5%, p=0.22). Despite these findings, the sample size might have been insufficient to detect differences in subgroup detection rates, such as lesion location; thus, further studies with larger cohorts are needed to elucidate these results. Additionally, even though the study was randomized, there were more PIRADS 5 cases in the TR group than in the TP group, which could have influenced the outcomes. A meta-analysis involving 8,826 patients demonstrated a higher detection rate of CSPCa in the anterior region with TP PBx, both in a per-lesion analysis (p=0.03) and a per-patient analysis (p<0.001)( 5 ). In the present study's cohort, there was no statistically significant difference between TP and TR PBx CSPCa detection rates in anterior lesions.

In this study, TP PBx was histologically more informative than the TR PBx in both univariable and multivariable analysis. Greater cancer core length and percentage were evidenced in TP target biopsy. A multicenter study of 1293 patients that underwent MRI-targeted and systematic TR or TP PBx reported that downgrading at radical prostatectomy was associated with a TR approach, lower cancer core length and percent on systematic PBx (all p≤0.03) ( 31 ). On multivariable analysis, higher maximum cancer core length and TP PBx were associated with a lower rate of downgrading. This suggests that TP PBx could play a role in reducing overtreatment in PCa management, underscoring the importance of its greater histological informativeness. Additionally, as more experience with focal therapy for PCa develops, the importance of histologically detailed biopsies remains to be determined and may have implications for patient selection or eligibility for focal therapy in the future.

Although randomized trials have shown that rectal cleansing with iodine may decrease TR PBx infectious complications ( 32 ), more recent studies comparing TP versus TR PBx have not adopted this practice ( 7 , 8 ). Similarly, we opted not to perform povidone-iodine preparation before TR or TP PBx. Since complications, procedural time, and patient-experienced pain levels under local anesthesia are similar for both approaches and CSPCa detection rates are comparable, the choice between TR and TP PBx can be based on patient preference. However, TP PBx has the advantages of avoiding rectal perforation, not requiring antibiotic prophylaxis in most patients, and potentially being more histologically informative with superiority for anterior lesions ( 27 ). The next challenge is to widely implement and increase training in the TP approach across academic and community centers worldwide, ultimately providing a more patient-centered approach for prostate biopsies.

This study has limitations. This is a single-center experience, therefore limiting the generalizability of the results. Nonetheless, the diverse patient population and the large cohort are a strength of this study. Although the data was collected on a prospectively maintained PBx database, this is a retrospective and non-randomized study. Nevertheless, possible selection bias or confounders were addressed by using multivariable logistic and linear regression models. Overall, the present study's results resonate with the conclusions reported in the clinical trials ( 7 , 9 , 10 ). However, this study evidenced that Black Race is an independent predictor for CSPCa detection, irrespective of the PBx approach, which wasn't demonstrated in prior trials. Additionally, the data support that TP PBx is histologically more informative than the TR PBx.

## CONCLUSIONS

In a large, diverse, multiracial and ethnic cohort, Black race was an independent predictor for CSPCa detection, but neither the biopsy approach nor anterior lesions on MRI were independent predictors. TP and TR PBx yielded similar CSPCa and high-grade PCa detection rates. TP PBx was histologically more informative, providing greater cancer core length and percent involvement on target biopsy samples.

## ABBREVIATIONS

ADC: = apparent diffusion coefficient
CSPCa: = Clinically significant cancer
DCE: = dynamic contrast-enhanced
DRE: = digital rectal examination
DWI: = diffusion-weighted imaging
GG: = Grade Group
ISUP: = International Society of Urological Pathology
mpMRI: = Multiparametric MRI
MRI: = magnetic resonance imaging
PBx: = prostate biopsy
PCa: = prostate cancer
PIRADS: = Prostate Imaging-Reporting and Data System
PSA: = Prostate specific antigen
PSAD: = Prostate specific antigen density
PV: = Prostate volume
SB: = Systematic biopsy
T2W: = T2-weighted
TB: = Target biopsy
TP: = Transperineal
TR: = Transrectal
TRUS: = Transrectal ultrasound
